# Coping with Uncertainty: Woodpecker Finches (*Cactospiza pallida*) from an Unpredictable Habitat Are More Flexible than Birds from a Stable Habitat

**DOI:** 10.1371/journal.pone.0091718

**Published:** 2014-03-17

**Authors:** Sabine Tebbich, Irmgard Teschke

**Affiliations:** 1 Department of Behavioural Biology, University of Vienna, Vienna, Austria; 2 Department of Behavioural Ecology and Evolutionary Genetics, Max Planck Institute for Ornithology, Seewiesen, Germany; CNRS, Université de Bourgogne, France

## Abstract

Behavioural flexibility is thought to be a major factor in evolution. It may facilitate the discovery and exploitation of new resources, which in turn may expose populations to novel selective forces and facilitate adaptive radiation. Darwin's finches are a textbook example of adaptive radiation. They are fast learners and show a range of unusual foraging techniques, probably as a result of their flexibility. In this study we aimed to test whether variability of the environment is correlated with flexibility. We compared woodpecker finches from a dry area (hereafter, Arid Zone), where food availability is variable, with individuals from a cloud forest (hereafter, *Scalesia* zone) where food abundance is stable. As parameters for flexibility, we measured neophilia and neophobia, which are two aspects of reaction to novelty, reversal learning and problem-solving. We found no differences in performance on a problem-solving task but, in line with our prediction, individuals from the Arid Zone were significantly faster reversal learners and more neophilic than their conspecifics from the *Scalesia* zone. The latter result supports the notion that environmental variability drives flexibility. In contrast to our prediction, Arid Zone birds were even more neophobic than birds from the *Scalesia* Zone. The latter result could be the consequence of differences in predation pressure between the two vegetation zones.

## Introduction

Behavioural flexibility is an important component of phenotypic plasticity and enables animals to react to changes in the environment. It may expose behaviour and associated morphology to divergent selection and is therefore thought to be a driving factor in adaptive radiation [1], [2], [3], [4], [5], [6], [7]. Two important pathways to behavioural flexibility are mediated by learning, namely (i) applying existing behaviour to new situations and (ii) acquiring new behavioural patterns to solve a problem [8], [9]. The tendency to encounter novel situations is in turn influenced by the propensity to approach and explore unfamiliar environments or objects. Combined, these abilities are particularly useful for the discovery of resources and the subsequent acquisition of new behavioural patterns to exploit them. This in turn may expose populations to novel selective forces and facilitate adaptive radiation [1], [2], [6], [10]. The latter hypothesis is also known as the Behavioural Drive Hypothesis which emphasises the role of behaviour in adaptive diversification [1], [2], [10]. The Flexible Stem Hypothesis is also concerned with behavioural flexibility as a driver of adaptive radiation but is more specific about the source of behavioural flexibility that precedes radiation. It states that adaptive radiations occur when an exceptionally adaptable stem species encounters a new environment [6].

The Darwin's finches are not only a poster example of adaptive radiation, they are also exceptionally flexible and innovative [8]. They use a wide range of feeding behaviours that incorporate feeding techniques or foraging substrates that are unusual for passerines. For example, the woodpecker finch (*Cactospiza pallida*) uses twigs to probe arthropods out of tree holes [11], [12] whereas the sharp-beaked ground finch (*Geospiza difficilis*) pecks at the developing feathers of sea birds, draws blood and drinks it [13]. The latter also cracks large sea bird eggs by pushing them against rocks [14]. It has been suggested that this wide range of feeding adaptations in Darwin's finches is a result of their flexibility and allows them to persist in the inhospitable conditions of the Galápagos Islands [6], [8], [15]. Two possible non-mutually exclusive explanations can account for the unusual flexibility of Darwin's finches: (i) the ancestor of Darwin's finches was unusually flexible and (ii) environmental factors selected for enhanced flexibility [8]. The aim of the current paper is to investigate environmental influence on behavioural flexibility.

Conditions on the Galápagos Islands are particularly inhospitable at low elevations. Although the Galápagos Islands are situated on the equator, the climate is unusually dry and highly seasonal, with only a short, rainy season from January to April and a dry season for the remainder of the year. At low elevations annual rainfall is very low (0–300 mm) and the onset of the dry and wet season is unpredictable. In addition to seasonal climatic fluctuations, the climate is strongly influenced by irregular El Niño phenomena which cause heavy rainfall for an extended period and often severe droughts in the following year [16]. These droughts induce high mortality in finch populations [17], [18] and add an additional factor of environmental uncertainty. However, on large islands predictability and variability of the environment varies between distinct vegetation zones. These vegetation zones range from deserts to lush cloud forests [16] and are a result of differences in precipitation along an altitudinal gradient. Food availability for Darwin's finches is limited and highly variable in the dry coastal areas but more stable at higher elevations [19]. Behavioural flexibility should allow Darwin's finches to tolerate extended periods of food shortage during the dry season by leading them to discover and use novel food sources or facilitating their entrance into new habitats.

Learning is an important pathway to flexibility as it allows animals to adjust their behaviour to environmental changes. However, learning should only be adaptive, if learning rates are sufficiently higher than the rates of environmental change [20] and should therefore vary with environmental stability and predictability.

Empirical support for the relationship between learning ability, and environmental variability comes from within-species comparisons. In black-capped chickadees *(Poecile atricapillus)*, birds from higher latitudes had better spatial memory [21] and were faster in problem-solving [22] than birds from lower latitudes. Three-spined sticklebacks (*Gasterosteus aculeatus*) from ponds and rivers differed in the types of information they used to solve a maze task where they could learn body-centred information (turn left or right) and landmarks [23]. Pond fish relied more than river fish on visual landmarks, which are reliable cues for orientation only in the stable pond habitats. Furthermore, an experimental study on captive cichlid fish (*Simochromis pleurospilus*) showed that individuals which experienced a change in food regime early in life, outperformed fish kept on constant rations in a visual discrimination task later in life [24]. A growing body of literature also suggests that individuals that invade novel and anthropogenic habitats show lower levels of neophobia and higher levels of innovativeness than non-invading and suburban-living conspecifics [25], [26], [27] (e.g. in house sparrows, *Passer domesticus*, [28], [29] and common mynas, *Acridotheres tristis* [30]).

Darwin's finches are an ideal study system with which to test the influence of environmental variability on flexibility because several closely related species live in vegetation zones that differ strongly in variability and accessibility of their food supply [19]. This provides an opportunity to study the effect of environmental variability on flexibility both at the intra- and interspecific level.

In the current study, we tested the hypothesis that a variable environment selects for behavioural flexibility. To do this, we compared the flexibility and learning performance of woodpecker finches from two habitats that differ strongly in environmental stability and related insect food supply: the ‘Arid Zone’ near the coast and the so-called ‘*Scalesia* Zone’, a humid cloud forest at 500 m asl. The foraging ecology of the woodpecker finch has been studied in detail and we therefore know how annual variation affects the food supply of this species in the two different vegetation zones [31]. In the humid *Scalesia* Zone, food abundance is high and stable throughout the year. We measured insect abundance in the three feeding substrates most frequently used by the woodpecker finches and found no significant difference between the wet and the dry season. In the Arid Zone, insect abundance was significantly lower than in the *Scalesia* Zone and was significantly lower in the dry than in the wet season. In addition to this inter-annual variation, the onset of the rainy seasons is highly variable [31] which means that woodpecker finches living in these areas are exposed to a high degree of variability and unpredictability within and between years.

For our comparison, we chose three aspects of information processing that we considered important for reacting to changes in food abundance and accessibility and which are thus important components of behavioural flexibility [8], [9]: reversal learning, problem-solving and reaction to novelty. Reversal learning is a standard test to investigate behavioural flexibility and measures how quickly a subject learns that a previously successful strategy is no longer rewarded. This ability may be especially advantageous in complex or changeable environments [32].

The discovery of novel resources is more important in areas where food abundance is variable and the behavioural precondition that promotes such discoveries is a propensity to approach novel resources. The approach or avoidance of novel resources may be driven by two different motivational systems that are probably not on opposite ends of one behavioural continuum and may vary independently [33]: Neophilia, the attraction towards novel objects, and neophobia, the fear of novel objects [34]. Once novel resources are approached, problem-solving may be crucial in exploiting them. We therefore predicted that woodpecker finches from the Arid Zone should be faster in reversal learning and problem-solving and they should less neophobic and more neophilic than their conspecifics from the *Scalesia* Zone.

## General Methods

### Ethics Statement

Permission to conduct this study was granted by the Galápagos National Park and the Charles Darwin research station (Project PC-16-07, Permit Nr. PR.PT.P004.R02). As our experiments are purely appetitive, strictly non-invasive and based exclusively on behavioural tests, they are classified as non-animal experiments in accordance with the Austrian Animal Experiments Act (§ 2. Federal Law Gazette No. 501/1989). After testing, eight birds were held in long-term captivity (≥1 year) for breeding purposes related to conservation. All other birds were held for the minimum amount of time required to complete the experiments, and then released at their site of capture. To assess the impact of extended periods in captivity on the well-being of the birds and for conservation purposes, we radio-tracked some of the birds upon release over a ten-day to two-week long period. For 3 birds from the wet zone that had spent a year or more in captivity, we found hat these birds re-adjusted well, quickly resumed feeding and territorial behaviors such as singing and nest-building.

### Study area, subjects and housing

The study was carried out at the Charles Darwin Research Station on Santa Cruz Island in the Galápagos Archipelago, Ecuador from October 2007–March 2008 and September 2008–January 2009. A total of 18 woodpecker finches were mist-netted during the dry season using playback of conspecific song. Eight woodpecker finches were caught in the Arid Zone at the site “Garrapatero” and 10 birds in the *Scalesia* Zone around “Los Gemelos”. The two study areas are located about 15 km from each other. We have no information on whether woodpecker finches move between these two areas.

Z-linked microsatellite data were used for sexing of the birds from the *Scalesia* Zone. Only these birds were sexed because they were part of a captive breeding program. Nine birds were identified as males and the results for one bird were inconclusive. Eight of the 7 birds from the Arid Zone sang in captivity and were therefore identified as males. Males react more strongly to playback of their own song which explains the high proportion of males in our sample.

We have no reliable information on the age of the birds. Three birds from the Arid Zone had lighter beaks, but beak colouration turned out to be an unreliable indicator of age in Darwin's finches as it not only varies with age but also with the breeding season [35], [36]. Since all birds were caught between 4 and 9 months after the end of the previous breeding season, they must have been at least subadult.

Following capture, the birds were first kept in a small habituation cage (0.5×0.5×1 m) for a maximum of 5 days. Thereafter, the birds were kept in outdoor aviaries (2×1×2 m) and released at the site of capture after the experiments were finished. Aviaries were equipped with natural branches and an experimental table on which food, the apparatus and the novel objects were presented.

Birds were kept singly and visually isolated from each other on a diet of mashed hard-boiled egg, grated carrot, mixed with commercial bird food mix (Orlux). Additionally, the birds received fresh fruit and fresh moths. Subjects were kept at 100% of their free-feeding weights by monitoring weight every three days and adjusting each individual's diet accordingly. For this purpose birds were trained to hop onto an electronic scale.

Please see Table S1 of the electronic supplementary material for more information on experimental subjects.

### Problem-solving task: box opening task

The apparatus was a box made of opaque, white Perspex with a transparent lid mounted on a wooden platform (20/20 cm). The lid was hinged to the back edge of the box and overlapped the front edge of the box. The box could be opened by pushing the protruding lip of the lid upwards. Before testing, birds were habituated to the box by feeding from it once while it was open. Subjects were given a maximum of 6 sessions of 25 min, receiving up to 3 sessions per day. Testing was ended when a subject had opened the box and ate the reward. If a bird did not contact the box during a session, it was re-habituated to the box by feeding from the open box and the session was repeated. A bird was given up to 2 extra sessions with a baited, closed box upon failing to make contact with the box in any one session. All sessions were observed from behind a screen and recorded with a camcorder (JVC GZ-MG130EK). We scored the following variables from the video footage: “Latency to first contact with the box” in the first session (s), “Total length of testing” (s), “Success” (opening box and gaining access to the food reward), “Number of pecks to the sides of the box” and the “Number of pecks to the lid”. A compound variable called “Number of contacts” was created by summing all actions on the box.

### The reversal task

#### Training

During the course of a previous experiment, birds had learned to remove a white cardboard lid of a single baited box. The birds that were not successful in removing the lid in the previous experiment were trained with a shaping procedure. In the initial phase of this procedure, the lid covered the box only partially so that the birds could see the food reward in the box. In successive trials, the amount of coverage was increased until the birds had learned to remove the lid.

#### Testing

The apparatus consisted of 2 white boxes (3×2×2.3 cm) covered with coloured lids that were mounted 10 cm apart on a wooden base (20×20 cm). In each trial a food reward was placed in one of the feeders out of sight of the subject. The apparatus was placed onto the experimental table and birds were then allowed to remove one of the two lids. A transparent Perspex divider (29.5×21 cm) prevented the birds from removing the lid of both feeders within one trial because the bird could not hop directly to the other feeder. Once the bird had made a choice and either had fed from the baited box or removed the lid from the empty box, the experimenter removed the apparatus and re-baited it out of sight. Each trial lasted no longer than 5 min. If a bird did not make a choice within 5 min, the experimenter removed the apparatus and replaced it with a baited open box until the bird fed from it. If the bird did so within 5 min, testing was resumed. Otherwise, testing was stopped until the next session.

Experiments were conducted in the home aviaries of the birds and food was removed from their aviaries 2 hours before tests. The experimenter observed the trial from behind a screen.

This experiment consisted of two phases: an initial “acquisition phase” and a “reversal phase”. In the acquisition phase, subjects were given a choice between two lids of different colours (orange and blue), one of which was the rewarded stimulus. Individuals were tested daily in two sessions of 10 trials in which the correct side was pseudo-randomised and counterbalanced right and left. The number of trials in which the reward was presented consecutively on one side never exceeded 3, except in one case of a side bias correction procedure (see below). Once a subject met the learning criterion (see below) in the initial colour discrimination, the colour-reward contingency was reversed in the reversal phase. Half of the birds in each population were first rewarded with orange and the other with blue. We measured the number of trials needed and the proportion of errors made in attaining criterion. Birds were given a maximum of 140 trials to meet the success criterion in each phase. In order to reach criterion, an animal either had to choose correctly 10 times out of 10 within one block; or, alternatively, a bird had to respond correctly in 16 or more trials within two consecutive blocks of 10 trials, whereby the number of correct responses in both blocks had to be at least eight. Given that the probability of reaching this criterion by chance increases steadily with each additional block, we assessed the animals' performance by implementing a simulation of the animals' choice behaviour in Matlab (The MathWorks, MA). In each simulated trial the response was modelled as a Bernoulli experiment with equal success and failure probabilities; choice behaviour was simulated in blocks of 10 trials until the above-mentioned criterion was reached. By running this simulation repeatedly (1000000 repetitions), we obtained an estimate of the probability distribution of the number of blocks required to reach the criterion by chance. This distribution thus provided a baseline against which we could compare the birds' performance. In particular, p-values associated with each bird's performance were obtained by comparing their number of blocks to criterion to the probability distribution generated by our simulation.

Due to an error in the application of the learning criterion one bird (lightgreen) was stopped in the acquisition phase after 3 blocks, choosing correctly 7 and 8 times in the last two blocks, and two birds (metal and rosapink) were stopped after 10 and 13 blocks in the reversal phase; both chose 7 and 9 times correctly within their last two blocks respectively. While these birds were excluded from the main analyses, there is strong indication that the bird had learned the reward contingencies. An analysis including these birds did not change the results.

#### Side bias correction

We determined that a subject had developed a side bias when it chose the same side of the apparatus for 6 trials in a row. In this case, rewards were only placed on the non-preferred side in the following trials until the bird chose the non-preferred side once.

### Reactions to novelty

We measured neophilia and neophobia with two experimental approaches as described below. Testing started between 14–47 days after capture. The median latency to testing did not differ between the birds from the two vegetation zones (Arid Zone: 23.0 days, range 14–30; *Scalesia* Zone: 39.5 days, range 14–47; *U* = 27, *n_Ar_* = 8, *n_Sc_* = 10, *p* = 0.3).

### Neophobia: feeding near novel objects

Between one and 6 days before the first novel object test, control feeding latencies were determined once, by measuring the latency to feed from the familiar feeding dish on the experiment table when first given food in the morning. After establishing this baseline feeding latency, a novel object was placed in the home aviary next to the familiar feeding dish on the experiment table and the latency to start feeding was recorded. Birds were first tested with a blue and white tissue package (1.5×6.5×2.5 cm) and one to five days later, with a green, purple and black loudspeaker (7×7.5×10 cm) This order of presentation was determined randomly. The exposure to the first object could have influenced the reaction to the second object but it is unlikely that this introduced a bias that led to a systematic difference between birds from the two zones. Experiments took place between 6:30–7:00am which was the usual time of the morning feed. At that time, birds presumably had a high motivation to feed, which should then have been the main motivation to approach the feeding dish and should only have been opposed by neophobia. Therefore, feeding latencies near the novel objects should reflect relative neophobia levels [37].

To establish whether the novel objects elicited a neophobic reaction, we compared the control feeding latency to the latency to feed near the novel objects. The presence of the loudspeaker elicited a neophobic reaction, as birds from both zones fed significantly later when the loudspeaker was nearby than in the control trial (*Scalesia* Zone: *Z* = −2.5, *p* = 0.012; Arid Zone: *Z* = −2.6, *p* = 0.011, Table 1). However, when the tissue package was nearby, only birds from the Arid Zone significantly delayed feeding (*Scalesia* Zone: *Z* = −1.1, *p* = 0.29; Arid Zone: *Z* = −2.1, *p* = 0.035, Table 1). Therefore we only used the difference between the loudspeaker and the control feeding latency as measure for neophobia.

**Table 1:** Latencies (s) to feed in the control condition (familiar feeding dish) and when a tissue package or loudspeaker were present.

	Control		Tissue package		Loudspeaker		Loudspeaker –Control	
Zone	Median	Range	Median	Range	Median	Range	Median	Range
Arid	6	1–16	16	5–202	24	5–220	15	3–215
*Scalesia*	6	3–40	7	3–43	10	3–48	5	0–15

Median and range are given.

### Neophilia: approaching novel objects

In the morning after feeding, a novel object (red toy car, 11×3×4.5 cm) was placed on the experiment table for up to 30 min. Subjects were not food-deprived, so neophilia, not hunger, was assumed to be the driving motivation of approach and exploratory activity. The food bowl was removed from the aviary during testing. Latencies to hop on the perch above the experiment table, latency to hop onto the table itself and latency to touch the toy car were measured. The experiment was ended when a bird touched the car or when it exceeded the maximum session length of 30 min. All subjects that did not come to the perch or the table were assigned at the ceiling value of 1801 s.

### Motivation

Motivation can strongly influence measures of behavioural tests and some studies have attempted to control for this variable. For example Sol et al. [38] tested motivation by presenting a food item before each behavioural assay and measuring the time subjects took to take it. Our study did not include specific measurements of this variable. However, we were still able to test for motivational differences between birds from the two zones using approximations of motivation from other facets of our data. We used the following measures to approximate motivation: from the reversal task, we used the number of trials in which individuals did not remove either of the two lids within the first 5 min of a trial; from the problem-solving task, we used the number of contacts with the box and the latency to approach the box; and from the neophobia test, we used the latency to approach the feeding dish in the control trials.

### Statistical analysis

We used non-parametric statistics because sample sizes were too small to assume that the data were normally distributed. Small sample sizes were also the reason for the univariate analyses with only single explanatory variables [39]. We compared the performances of Arid Zone and *Scalesia* Zone birds in each task with Mann-Whitney *U*-tests. 8 birds from the *Scalesia* Zone and 8 birds from the Arid Zone participated in the reversal task. In all other experiments we tested 10 birds from the *Scalesia* Zone and 8 birds from the Arid Zone.

Responses to novel objects were compared with performances during one control situation using Wilcoxon signed ranks test. We used the Fisher's exact test to test for an association between zone and success in opening the box and/or between zone and touching the novel objects.

## Results

### Problem-solving task

Our measures of motivation did not differ for the birds from the two vegetation zones. We found no significant difference in the latency to first contact with the box (*U* = 35.0, *n_Ar_* = 8, *n_Sc_* = 10, *p* = 0.7) nor in the number of contacts they made with box (*U* = 33.0, *n_Ar_* = 8, *n_Sc_* = 10, *p* = 0.5). We also found no difference in the two measures of problem-solving abilities for the birds from the two vegetation zones: There was no association between success in opening the box within six sessions and zone (Arid Zone: 3 of 8 birds successful; *Scalesia* Zone: 5 of 10 birds successful; Fisher's Exact Test: *χ* ^2^ = 0.3, *p* = 0.7) nor was there a significant difference in the latency to success between zones (*U* = 32.5, *n_Ar_* = 8, *n_Sc_* = 10, *p* = 0.5, Table 2).

**Table 2:** Measured parameters of the box opening task for woodpecker finches from the Arid and the *Scalesia* Zone.

	Latency to contact (s)		Nr. of contacts		Latency to success (min)	
Zone	Median	Range	Median	Range	Median	Range
Arid	35	7–1500	277	12–483	150	1–201
*Scalesia*	20	6–992	225	74–1027	137	15–150

Median and range are given for latency to first contact, number of contacts with the box and latency to success (obtaining the reward).

### The reversal task

Birds were highly motivated to participate in this task and only failed to make a choice within 5 min in 6 out of 657 trials in the acquisition phase and in 7 trials out of 1520 in the reversal phase (all birds combined). Failure to make a choice was recorded 9 times for birds from the Arid zone and 4 times for birds from the *Scalesia* Zone (both phases combined).

In the acquisition phase 15 out of 16 birds reached the learning criterion within 140 trials and in the reversal phase 13 out of 14.

**Figure 1:**
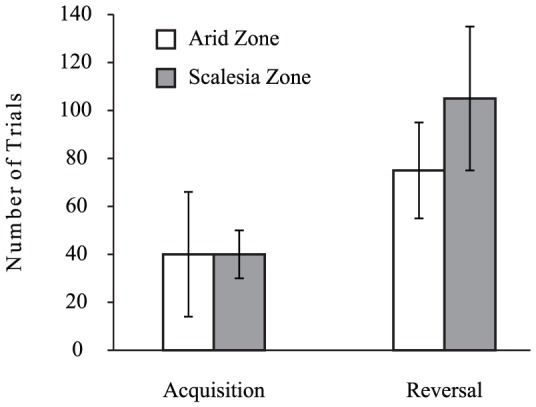
Number of trials that woodpecker finches from the Arid and *Scalesia* zones needed to reach the learning criterion in the acquisition and reversal phase of the colour discrimination task. Bars show medians and interquartile ranges.

Woodpecker finches from the *Scalesia* and the Arid Zone did not differ significantly in the number of trials needed to meet criterion in the acquisition phase. (*U* = 25.5, *n_Ar_* = 8, *n_Sc_* = 7, *p* = 0.78, Figure 1) or the proportion of errors made (*U* = 17.0, *p* = 0.23) in doing so (*Scalesia* Zone: median = 0.35, IQR = 0.15; Arid Zone: median = 0.31, IQR = 0.15). However, woodpecker finches from the Arid Zone needed significantly fewer trials to meet criterion in the reversal phase (*U* = 6.0, *n_Ar_* = 8, *n_Sc_* = 6, *p* = 0.02, Figure 1) though the two populations did not differ in the proportion of errors made in the reversal phase (*Scalesia* Zone: median = 0.61, IQR = 0.18; Arid Zone: median = 0.55, IQR = 0.16; *U* = 17.0, *p* = 0.41).

### Neophobia

Woodpecker finches from the two zones did not differ in the latency to feed in the control trials (*U* = 31.0, *n_Ar_* = 8, *n_Sc_* = 10, *p* = 0.4, Table 1).

The difference between the latencies to feed near the loudspeaker and the control feeding latency was significantly larger for birds from the Arid Zone than for birds from the *Scalesia* Zone (*U* = 10.0, *n_Ar_* = 8, *n_Sc_* = 10, *p* = 0.006). Thus woodpecker finches from the Arid Zone showed higher levels of neophobia.

### Neophilia

Six of 8 woodpecker finches from the Arid Zone but only 2 of 10 woodpecker finches from the *Scalesia* Zone touched the toy car in their aviary within 30 min (Fisher's Exact Test: *χ* ^2^ = 0.281, *p* = 0.053). Thus, this result fell just short of significance. The latency to touch the car was significantly longer for woodpecker finches from the *Scalesia* Zone (median = 1801 s, IQR = 418) than for birds from the Arid Zone (median = 84 s, IQR = 1393; *U* = 15, *p* = 0.027).

## Discussion

In our study we aimed to provide support for the hypothesis that environmental variability selects for behavioural flexibility. We predicted that woodpecker finches from the variable Arid Zone should be faster in reversal learning and problem-solving and that, when encountering novel objects, they should be more neophilic but less neophobic than their conspecifics from the stable *Scalesia* Zone. In accordance with our predictions, birds from the Arid Zone were faster in reversal learning and more neophilic. Contrary to our predictions, we found no difference between birds from the two zones in problem-solving ability and Arid Zone birds were actually more neophilic than woodpecker finches from the *Scalesia* Zone.

Our study is the first to find a difference in reversal learning between individuals from a stable and a variable environment. Because reversal learning entails flexible responding to a fixed set of stimuli based on a fluctuating reward regimen, it closely mimics the demands of a fluctuating environment [32]. In line with our prediction, birds from the Arid Zone learned about the reversal of reward contingencies significantly faster than their conspecifics from the *Scalesia* Zone. Previous studies have demonstrated a positive relationship between reversal learning and environmental complexity or variability at the interspecific level: Bond et al. [32] compared three corvid species whose environment varied in social and ecological complexity in a serial reversal learning task. They found that the species from the most complex social environment was most flexible and not the species with the most generalist ecology. Day et al. [40] found differences in reversal learning in two congeneric lizards with different ecologies. However, interspecific comparisons have only limited explanatory power as species differences in morphology and other ecological variables can influence the behavioural outcome [41]. The influence of these contextual variables cannot be excluded, but their impact may be diminished by comparing populations of the same species that vary in a trait of interest. To date, only two studies have looked at population differences in learning in relation to environmental variability [21], [22]: In black-capped chickadees (*Poecile atricapillus*), birds from a harsh environment were faster to feed from a novel feeding dish and also faster to solve an operant task [22]. This contrasts with our study which shows that woodpecker finches from the Arid Zone were more neophobic and not faster in learning a problem-solving task than birds from the *Scalesia* Zone. One possible reason for the discrepancy in neophobia between our study and that of Roth et al. [22] is the diversity of factors that select for certain novelty reactions.

Reaction to novelty is thought to be influenced by two motivational systems, the attraction and the aversion to novelty, which in turn may be driven by different selective pressures [33]. Neophilia—the attraction to novelty—is thought to be beneficial, especially in a harsh and variable environment, since it can facilitate the discovery of new resources, help in finding new patches of familiar resources or in developing new means of obtaining familiar resources. However, neophilia also has costs, such as the risk of predation and neophobia may have evolved to inhibit the costs of neophilia [33]. Thus the balance between neophilia and neophobia depends on the ecological conditions: In environments where the likelihood of and the benefits arising from encountering novel food resources are high and predation pressure is low, selection should favour approach and exploration of novel resources, whereas in environments with high predation pressure and low encounter rates with unfamiliar resources, avoidance should be more beneficial [30].Our finding that woodpecker finches from the Arid Zone tended to be more neophilic than those from the *Scalesia* zone could be explained by the higher benefits of encountering novel resources in the habitat with variable food supply. In contrast, the higher neophobia of the woodpecker finches from the Arid zone could be driven by the cost of exploration, such as that imposed by predation. For Darwin's finches, we only have information about the variability of the environment in the two vegetation zones but none on predation pressure.

The source of difference in learning and reaction to novelty between woodpecker finches from the two vegetation zones could be due to genetic differences as shown by Roth et al.'s [22] common garden experiment on black-capped chickadees, or to different influences during ontogeny, as shown in a cichlid species by Kotrschal & Taborsky [24], or a combination of both. Only further common garden studies can reveal the source for the difference in the two populations of woodpecker finches.

The question of whether the harsh and variable environment of the Arid Zone is also an important driver for the high frequency of feeding innovations and thus a driver for the adaptive radiation in Darwin's finches cannot be clearly answered with our data set. On the one hand, the Arid Zone birds are good reversal learners which may reflect innovative capacity as several studies have found a significant correlation between reversal learning performance and rates of foraging innovation across species [26], [42], [43], but see [32].

It was surprising to find that woodpecker finches from the Arid Zone were not faster or better at solving the problem-solving task since it is thought that success on such tasks correlates with proficiency in exploiting novel resources and thus important aspects of innovation [9]. However, only a low number of woodpecker finches were able to open the box (8 out of 18 woodpecker finches from both zones), indicating that the task may have been too difficult to render meaningful results.

Overall, our results must be interpreted with caution as we only compared two populations and sample sizes were small. The small sample size also limited analysis to non-parametric comparisons with only one explanatory variable per test because, with our data set of ≤10 subjects per zone, the number of cases within subgroups of zone would have been too small to yield reliable results [39]. Thus, factors like zone and motivation could not be integrated into a parametric analysis, incorporating multiple explanatory variables. Since we had no information on the age of our test subjects or their colour preferences we cannot exclude an influence of these variables on our data. Again, because of our small sample size and the variation in the number of days before testing neophobia (14–47 days), we also cannot exclude an influence of variation on the novelty reactions we observed.

However, we can assert confidently that, compared to other passerines, woodpecker finches from both zones are very fast in reversal learning and in solving novel tasks [8], [44], [45]. This supports the idea that variability of the environment may not be the only factor driving feeding innovations in Darwin's finches but favours a multi-factor scenario. In this scenario, a flexible ancestor, reduced predation pressure and other environmental factors associated with living on islands may have contributed to the evolution of the exceptional array of feeding innovation found in this species group.

## Supporting Information

Table S1 **History and sex of experimental subjects.**
(DOC)
