# Supplementary material for: Coping with Uncertainty: Woodpecker Finches (Cactospiza pallida) from an Unpredictable Habitat Are More Flexible than Birds from a Stable Habitat
Source: PLoS One. 2014 Mar 17;9(3):e91718. doi: 10.1371/journal.pone.0091718 (PMC3956741; doi:10.1371/journal.pone.0091718)
Supplement: Table S1 — History and sex of experimental subjects. (DOC) [file pone.0091718.s001.doc]

Table S1: History of experimental subjects

| Subject | Zone of capture | Capture date | Beak colour | Sex |
| --- | --- | --- | --- | --- |
| blackblue | Arid | 31.01.2008 | dark | M |
| blackgreen | Arid | 25.09.2008 | dark | M |
| bluered | Arid | 18.01.2008 | dark | unknown |
| greengreen | Arid | 22.09.2008 | light | M |
| orangeblue | Arid | 03.02.2008 | dark | M |
| purpleblack | Arid | 24.09.2008 | dark | M |
| purplepink | Arid | 24.09.2008 | light | M |
| redblack | Arid | 29.01.2008 | dark | M |
| blackpink | *Scalesia* | 21.11.2007 | dark | M |
| blueblue | *Scalesia* | 20.12.2007 | dark | M |
| lightgreen | *Scalesia* | 31.10.2007 | dark | M |
| metal | *Scalesia* | 20.11.2007 | dark | M |
| orangegreen | *Scalesia* | 19.11.2007 | dark | M |
| purplegreen | *Scalesia* | 21.12.2007 | dark | M |
| redgreen | *Scalesia* | 31.10.2007 | dark | M |
| redL | *Scalesia* | 26.10.2007 | dark | unknown |
| rosablue | *Scalesia* | 19.11.2007 | dark | M |
| rosapink | *Scalesia* | 31.10.2007 | dark | M |
